# Antiviral Properties of *Moringa oleifera* Leaf Extracts against Respiratory Viruses

**DOI:** 10.3390/v16081199

**Published:** 2024-07-25

**Authors:** Rosa Giugliano, Valeria Ferraro, Annalisa Chianese, Roberta Della Marca, Carla Zannella, Francesca Galdiero, Teresa M. A. Fasciana, Anna Giammanco, Antonio Salerno, Joseph Cannillo, Natalie Paola Rotondo, Giovanni Lentini, Maria Maddalena Cavalluzzi, Anna De Filippis, Massimiliano Galdiero

**Affiliations:** 1 Department of Experimental Medicine, University of Campania “Luigi Vanvitelli”, 80138 Naples, Italy; rosa.giugliano@unicampania.it (R.G.); annalisa.chianese@unicampania.it (A.C.); roberta.dellamarca@unicampania.it (R.D.M.); carla.zannella@unicampania.it (C.Z.); francescagaldiero21@gmail.com (F.G.); anna.defilippis@unicampania.it (A.D.F.); 2 Department of Pharmacy—Drug Sciences, University Aldo Moro-Bari, Via Orabona 4, 70126 Bari, Italy; valeria.ferraro@uniba.it (V.F.); nataliepaola.rotondo@uniba.it (N.P.R.); giovanni.lentini@uniba.it (G.L.); mariamaddalena.cavalluzzi@uniba.it (M.M.C.); 3 Department of Health Promotion, Mother and Child Care, Internal Medicine and Medical Specialties, University of Palermo, 90127 Palermo, Italy; teresa.fasciana@unipa.it (T.M.A.F.); anna.giammanco@unipa.it (A.G.); 4 Forza Vitale, Via Castel del Monte, 194/C, 70033 Corato, Italy; info@forzavitale.it (A.S.); info@forzavitale.it (J.C.)

**Keywords:** *M. oleifera*, antiviral activity, microwave-assisted extraction, natural products, HPLC, respiratory viruses, coronavirus, measles virus

## Abstract

*Moringa oleifera* (*M. oleifera*) is a plant widely used for its beneficial properties both in medical and non-medical fields. Because they produce bioactive metabolites, plants are a major resource for drug discovery. In this study, two different cultivars of leaves of *M. oleifera* (Salento and Barletta) were obtained by maceration or microwave-assisted extraction (MAE). We demonstrated that extracts obtained by MAE exhibited a lower cytotoxic profile compared to those obtained by maceration at concentrations ranged from 25 to 400 µg/mL, on both Vero CCL-81 and Vero/SLAM cells. We examined their antiviral properties against two viruses, i.e., the human coronavirus 229E (HCoV-229E) and measles virus (MeV), which are both responsible for respiratory infections. The extracts were able to inhibit the infection of both viruses and strongly prevented their attack and entry into the cells in a range of concentrations from 50 to 12 µg/mL. Particularly active was the variety of Salento that registered a 50% inhibitory concentration (IC50) at 21 µg/mL for HCoV-229E and at 6 µg/mL for MeV. We identified the presence of several compounds through high performance liquid chromatography (HPLC); in particular, chlorogenic and neochlorogenic acids, quercetin 3-O-β-d-glucopyranoside (QGP), and glucomoringin (GM) were mainly observed. In the end, *M. oleifera* can be considered a promising candidate for combating viral infections with a very strong action in the early stages of viral life cycle, probably by destructuring the viral particles blocking the virus–cell fusion.

## 1. Introduction

Respiratory tract infections (RTIs) are one of the leading causes of mortality worldwide, registering more than 4 million deaths per year [1,2]. Their rapid spread and seasonality cause frequent annual epidemics among the world’s population. The symptoms can be mild or severe and, in some cases, even fatal, especially in children, the elderly, and immunocompromised people [3]. The clinical picture may worsen as a consequence of the possible mixed etiology with viruses and pathogenic bacteria co-detected in respiratory secretions. Around 90% of upper respiratory tract infections (URTIs) are caused by viruses, while only 10% are due to bacteria [4], as also evidenced by the recent COVID-19 outbreak. Here, severe complications, such as pulmonary inflammation, lung tissue damage, edema, and the exacerbation of inflammatory processes, have been found often coupled with secondary bacterial infections, such as those caused by *Acinetobacter baumannii*, *Streptococcus pneumoniae*, *Pseudomonas aeruginosa,* and *Staphylococcus aureus* [5]. However, many studies also emphasize the co-presence of several respiratory viruses, including respiratory syncytial virus (RSV), rhinovirus (RV), influenza virus (IV), parainfluenza virus (PIV), coronavirus (CoV), and adenovirus (AdV) [2,6,7,8]. Respiratory viruses are classified into non-influenza and influenza viruses [9]. The first group includes CoVs, which are enveloped viruses with (+) ssRNA belonging to the *Coronaviridae* family. There are seven human coronaviruses subdivided into the genera alphacoronavirus (229E and NL63) and betacoronavirus (OC43, HKU1, severe acute respiratory syndrome coronavirus type 1 and 2, and Middle East respiratory syndrome coronavirus) [10]. Usually, CoV infections are characterized by mild symptomatology, with flu symptoms and cold, but there are three highly pathogenic species listed above that have caused either pandemics or deadly epidemics worldwide, i.e., the severe acute respiratory syndrome coronavirus (SARS-CoV) in 2002, the Middle East respiratory syndrome coronavirus (MERS-CoV) in 2012, and the recent pandemic (SARS-CoV-2), which arose in December 2019 [11]. Other noteworthy viruses affecting the respiratory tract are PIVs, which remain one of the main causes of pediatric hospitalizations. PIVs are enveloped negative-sense single-stranded RNA viruses belonging to the *Paramyxoviridae* family, like the measles virus (MeV); they are highly contagious and transmitted via respiratory droplets. All paramyxoviruses initially infect the upper respiratory tract and subsequently reach the lower airways [12]. The COVID-19 pandemic has led to a decline in vaccination and surveillance services worldwide, and as a result, in 2022, measles cases in the world increased by 18%, with 43% of cases resulting in death. There was an estimated number of 9 million cases and 136 thousand deaths, especially among children. In this context, there is an urgent need to discover and use new antiviral agents and approaches.

Since ancient times, the therapeutic properties of plants have been recognized for the treatment of pathological conditions and represent an important tool for the identification of new natural drugs. About 25% of all drugs approved by the Food and Drug Administration (FDA) and by the European Medical Agency (EMA) derive from plants [13]. *Moringa oleifera* (*M. oleifera*), native to India and Africa, is a fast-growing plant belonging to the *Moringaceae* family [14]. The genus *Moringa* contains 13 different species and, among these, *M. oleifera* is the most widespread in tropical and subtropical areas [15]. Commonly known as “miracle tree”, “wonder tree” or “tree of life”, it is used as a nutritional food in various parts of the world and as a remedy due to its many benefits [16,17,18]. Biological functions have been reported from all parts of the plant, including its seeds, leaves, fruits, flowers, and roots. In particular, the leaves are rich in vitamins, proteins, and minerals, while the seeds contain a considerable amount of oil. Additionally, *M. oleifera* is rich in phytochemicals such as flavonoids, alkaloids, tannins, and saponins, providing antioxidant and antimicrobial effects [19]. For its properties, it has aroused much interest and is a good candidate in nutraceutical applications, as well as in the cosmeceutical, agricultural, and pharmaceutical sectors [20,21]. Because it is a safe plant, researchers have also developed several phytopharmaceutical formulations based on *M. oleifera*, such as ointments, creams, and nasal sprays for allergies and inflammation [22,23,24]. Several studies have analyzed the antiviral properties of *M. oleifera*, reporting a virucidal effect against Newcastle disease virus, varicella-zoster virus, human immunodeficiency virus (HIV), hepatitis B virus, and influenza A virus [25,26]. Herein, we evaluate for the first time the antiviral activity of *M. oleifera* against two viruses responsible for respiratory tract infections: the human coronavirus 229E (HCoV-229E) and MeV.

## 2. Materials and Methods

### 2.1. Moringa Leaf Extract Preparation

The leaves of two different varieties of Apulian *M. oleifera* were used for this work. *Moringa* leaves from plants cultivated in a greenhouse in Lecce province (Puglia, Italy) were harvested in 2023. *Moringa* leaves from plants cultivated in an open field in the Barletta area (Puglia, Italy) were harvested in 2022. After drying, *Moringa* leaves underwent two different extraction procedures, namely microwave-assisted extraction (MAE) and maceration, using H_2_O, 50% EtOH, and 70% EtOH as extracting solvents. They were evaporated, dissolved in PBS 1X at a concentration of 10 mg/mL, and tested against viruses. The extracts obtained were named with the following abbreviations: MwS (Salento cultivar extracted by microwave), MwB (Barletta cultivar extracted by microwave), MaS (Salento cultivar extracted by maceration), and MaB (Barletta cultivar extracted by maceration). The numbering indicates the solvent used: 1 (extraction in water), 2 (extraction in 50% EtOH), and 3 (extraction in 70% EtOH). All information is summarized in the following Table 1.

#### 2.1.1. Conventional Extraction

A total of 2.0 g of *Moringa* leaf powder was poured into 20 mL of the appropriate solvent, and the mixture was stirred at room temperature for 48 h. The solution was then filtered, and the solvent was evaporated under vacuum. The sample was stored at −20 °C until used.

#### 2.1.2. Microwave-Assisted Extraction (MAE)

A closed-system MAE was carried out in a CEM Discover Bench Mate microwave reactor equipped with Synergy software, working at a constant temperature and with continuous stirring. The temperature was measured and controlled by a built-in infrared detector. Briefly, 200 mg of leaf powder was poured into 2 mL of the appropriate solvent [*w*/*v*, 1:10], and the mixture was irradiated with microwaves at 80 °C for 5 min. The solution was then filtered, and the solvent was evaporated under vacuum or lyophilized. The sample was stored at −20 °C until used. The yields obtained for each sample are reported in Table 2.

### 2.2. Cell Lines and Viruses

Cell lines used in the present study were Vero CCL-81 (American-type culture collection, ATCC, Manassas, VA, USA) and Vero/hSLAM cells (ECACC 04091501, Porton Down, UK), both renal epithelial cells of African green monkey. In addition, for the assays on human cells, A549 cell line (ATCC, CCL-185) was used. Cells were cultivated in Dulbecco’s Modified Eagle Medium (DMEM) (Microgem, Naples, Italy) with 4.5 g/L glucose, 2 mM l-glutamine, and 100 IU/mL penicillin-streptomycin solution, supplemented with 10% fetal bovine serum (FBS) (Microgem, Naples, Italy). The cell lines were maintained in a humidified atmosphere with 5% CO_2_ at 37 °C. Measles virus (ATCC VR-24) and HCoV-229E (ATCC VR-740) were purchased from ATCC and propagated and titrated on Vero/hSLAM cells and Vero CCL-81 cells, respectively.

### 2.3. Cytotoxicity Assay

Cytotoxicity assays were achieved in 96-well culture plates and determined by [3-(4,5-dimethylthiazol-2-yl)-2,5-diphenyltetrazolium bromide] (MTT) measurements. (Sigma-Aldrich, St. Louis, MO, USA). Briefly, 2 × 10^4^ cells/mL VERO/hSLAM or Vero CCL-81 cells were seeded in a 96-well plate, and, the next day, they were treated in triplicate with different concentrations of extracts ranging from 25 to 400 µg/mL for 24 h (h). Then, 100 μL of MTT solution (5 mg/mL) was added to each well and incubated for 3 h at 37 °C to allow the reduction of MTT solution from yellow to blue in metabolically active vital cells [27]. Finally, the plate was emptied, and 100 µL of DMSO (100%) was added to each well to dissolve the formazan crystals. The untreated cells were used as a positive control, while the cells treated with 100 µL of DMSO (100%) represented the negative control; PBS was added as solvent control. Lastly, cell viability was calculated by reading the absorbance at 570 nm with a microplate reader.

### 2.4. Antiviral Assay

The antiviral activity was evaluated via plaque reduction assay, a consolidated method whose output is plaque count following infected cell lysis. Practically, Vero CCL-81 or Vero/hSLAM cells (1.3 × 10^5^ cells/well) were seeded in 24-well plates and, the next day, they were treated with the extracts at concentrations ranging from 3 to 200 µg/mL. Four different experiments were conducted to understand the stage of the viral cycle in which the extracts acted. In each test, after the virus adsorption time, the supernatant was removed, and the cells were washed with citrate buffer for 5 min. The cell monolayer was covered with fresh culture medium supplemented with 5% carboxymethylcellulose (CMC) (Sigma-Aldrich, Darmstadt, Germany) [28]. After 48 h incubation, the plate was emptied and washed with PBS. The cells were fixed in 4% formaldehyde for 20 min and then stained with 0.5% crystal violet. The percentage of viral inhibition was calculated by comparing the number of plaques observed in treated samples, with the negative control (CTRL−) represented by the infected and untreated cells. As positive control (CTRL+), the extract of the alga *Galdieria sulphuraria* was used at 50 µg/mL against HCoV-229E [29]; meanwhile, the peptide AR-23 was used at 25 µg/mL against MeV [30]. Four treatments differed in the time of addition of the substance and were performed as follows:

- Co-treatment assay: extracts, at selected concentrations, and virus at multiplicity of infection (MOI) of 0.01, were mixed in a 1:1 ratio and added simultaneously to the cell monolayer for the time of viral adsorption;
- Virus pre-treatment assay: the viral suspension, containing 10^4^ PFU, was preincubated with extracts for 1 h at 37 °C. Then, the mixture (extract and virus) was diluted and dispensed on the cell monolayer for the time of viral adsorption;
- Cell pre-treatment assay: the cell monolayer was first treated with extracts for 1 h; then, cells were covered with viral suspension for the time of viral adsorption;
- Post-infection assay: the cell monolayer was first infected with the virus at the time of viral adsorption. Then, the cells were washed and treated with extracts for 1 h.

Moreover, two temperature-shift assays were conducted to differentiate viral attachment and penetration.

(a) Attachment assay: cells were seeded at an initial density of 1.3 × 10^5^ cells/well in a 24-well plate and incubated at 37 °C overnight to obtain a monolayer. The next day, the cells were pre-cooled at 4 °C for 30 min and co-treated with the virus (MOI = 0.01) and extracts at 4 °C for the time of viral adsorption. Then, the supernatant was removed, and the monolayer was washed. The plate was filled with CMC and incubated at 37 °C for 48 h.
(b) Entry assay: the cells were plated as described above and pre-cooled to 4 °C for 30 min. The cells were infected with the virus (MOI = 0.01) and incubated at 4 °C for the time of adsorption. After removing the supernatant, the cells were washed, treated with the extracts, and incubated at 37 °C for 1 h. At the end of treatment, the plate was filled with CMC and incubated at 37 °C for 48 h.

### 2.5. High-Performance Liquid Chromatography (HPLC) Investigation

HPLC analysis was performed by a Varian ProStar HPLC system equipped with Solvent Delivery Module model 230, Autosampler model 410, and PDA Detector model 330. All samples were filtered before injection using disposable syringe filters Chromafil Xtra RC-45/25. Commercially available chlorogenic and neochlorogenic acids, quercetin-3-O-β-d-glucopyranoside, and glucomoringin were used as reference standards Chromatographic separations were performed in different conditions, based on the analyte to be identified, as described below. Chlorogenic and neochlorogenic acids: column Luna Phenomenex C18 (250 × 4.6) 5 µm, mobile phase ACN/H_3_PO_4_ 0.5% (11.5/88.5), injection volume 10 µL, flow rate 1 mL/min, wavelength 327 nm, column temperature 25 °C; Quercetin-3-*O*-β-d-glucopyranoside: column Luna Phenomenex C18 (150 × 4.6) 5 µm, mobile phase H_3_PO_4_ 0.3%/MeOH/ACN (55/35/10), injection volume 5 µL, flow rate 1 mL/min, wavelength 254 nm, column temperature 30 °C; Glucomoringin: column Luna Phenomenex C18 (250 × 4.6) 5 µm, mobile phase TFA 0.1%/ACN (95/5), injection volume 10 µL, flow rate 1 mL/min, wavelength 230 nm, column temperature 30 °C.

### 2.6. High-Performance Thin Layer Chromatography (HPTLC) Analysis

For HPTLC analysis, a mobile phase consisting of ethyl acetate:formic acid:acetic acid:water (100:11:11:26) was used. The application of standards and samples was performed using a semimicro applicator (Cellogel Electrophoresis Co., Milan, Italy). Approximately 1.5 μL of extract sample and 1.5 μL of 1 mg/mL solution of chlorogenic acid (CA), neochlorogenic acid (NCA), quercetin 3-*O*-β-d-glucopyranoside (QGP), glucomoringin (GM), and quercetin dihydrate (Q) standards were separately applied in the form of bands (1.5 μL × 8 mm) at 1 cm from the bottom using a TLC Silica gel 60 F254 pre-coated plate (Merck). The plate was developed up to the distance of 8 cm from the bottom, air dried, heated at 100 °C for 5 min, sprayed with 1% (*w*/*v*) diphenylboryloxyethylamine in methanol (NP), then sprayed with 5% (*w*/*v*) polyethylene glycol 4000 (PEG4000) in ethanol, air dried, and visualized by viewing in UV-cabinet under long wavelength (366 nm) [31]. All solvents are HPLC grade and were purchased from Merck, Honeywell, and ND J.T. Baker; chlorogenic acid was purchased from Sigma-Aldrich; neochlorogenic acid from Merck; and glucomoringin and quercetin-3-*O*-β-d-glucopyranoside from Extrasynthese.

### 2.7. Statistical Analysis and Selectivity Index Calculation

Graphs, the 50% cytotoxic concentration (CC50), the 50% inhibitory concentration (IC50), and statistical analysis were performed with GraphPad Prism ver. 8.2.1 (GraphPad Software, San Diego, CA, USA, www.graphpad.com (accessed on 12 March 2024)). The selectivity index (SI) has been calculated from the cytotoxicity and inhibitory data (CC50/IC50).

## 3. Results

### 3.1. Cytotoxicity

**Figure 1:**
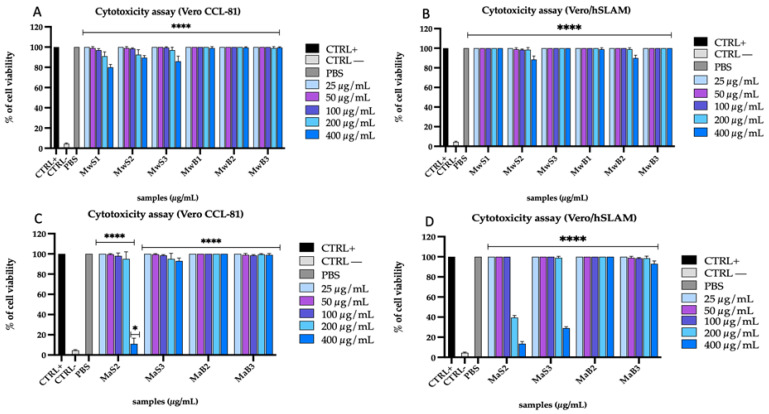
Cytotoxicity of *M. oleifera* extracts obtained by MAE (**A**,**B**) and maceration (**C**,**D**) on Vero CCL-81 and Vero/hSLAM cell lines. Positive control (CTRL+) was represented by untreated cells, negative control (CTRL−) by cells treated with DMSO and PBS was used as a vehicle. Two-way ANOVA was utilized for statistical analyses. Dunnett’s test was utilized for multiple comparisons. **** *p* < 0.0001, * *p* < 0.021, ns: not statistically significant.

The toxicity of the extracts of both cultivars obtained with two different extraction methods, namely MAE (Mw) and maceration (Ma) extraction, was evaluated on two cellular models, i.e., Vero CCL-81 and Vero/hSLAM, via the MTT test. The colorimetric reaction due to the reduction of tetrazolium salts in formazan allowed the measurement of viable cells expressed as percentages (Figure 1).

The extracts obtained by maceration showed no cytotoxicity on Vero CCL-81 cells except for MaS2, which recorded 90% toxicity at the highest tested concentration (400 µg/mL); similarly, the extract exhibited 90% and 60% toxicity at 400 µg/mL and 200 µg/mL, respectively, on Vero/hSLAM cells. On the other hand, the extracts obtained from the MAE (MwS and MwB) method, did not cause a toxic effect at the concentrations examined on both cell lines. In addition, the solvent in which the samples were dissolved (PBS) did not induce any effect on cell viability, as shown in Figure 1. Altogether, these data indicate that the MAE method is safer than maceration since MAE extracts exhibited no cytotoxicity on the two cellular models used in the present study.

### 3.2. Inhibitory Activity of M. oleifera Extracts by Plaque Reduction Assay

The antiviral activity of the different extracts of *Moringa* was investigated against two respiratory viruses: the coronavirus HCoV-229E and the paramyxovirus MeV.

#### 3.2.1. Antiviral Activity against HCoV-229E

**Figure 2:**
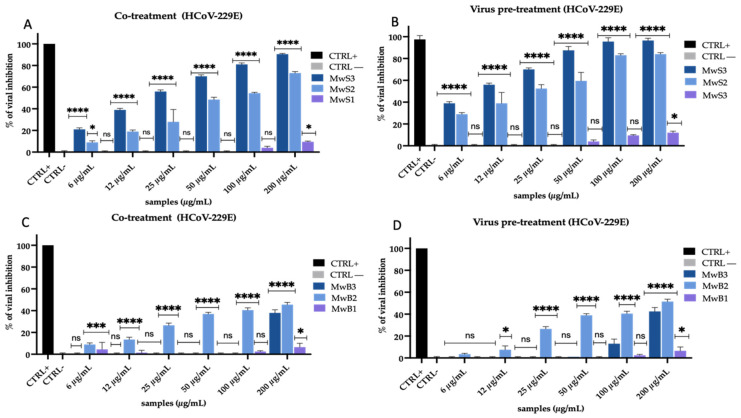
Antiviral activity of *M. oleifera* extracts against HCoV-229E. Co-treatment test (**A**) and virus pre-treatment (**B**) of MwS extracts; co-treatment test (**C**) and virus pre-treatment test (**D**) of MwB extracts. Algal extract at 50 µg/mL was used as a positive control (CTRL+), while infected and untreated cells represented negative control (CTRL−). Two-way ANOVA was used for statistical analysis. The Dunnett test was used for multiple comparisons. **** *p* < 0.0001, *** *p* ≤ 0.0002, * *p* < 0.0332, ns: not statistically significant.

To assess whether and which extracts of *M. oleifera* had an antiviral effect against HCoV-229E, Vero CCL-81 cells were co-treated for 1 h with each extract, at different non-toxic concentrations (from 6 to 200 µg/mL), and with viral suspension (co-treatment test, Figure 2A,C).

Among the MwS extracts (Figure 2A), MwS3 and MwS2 showed very significant rates of viral inhibition with a dose-dependent trend, while MwS1 was ineffective. In detail, MwS3 and MwS2 exhibited an IC_50_ at 21 μg/mL and 50 μg/mL, respectively. On the other hand, the MwB extracts (Figure 2C), did not show a strong activity compared to the cultivar of Salento. In fact, at the highest concentration assessed (200 µg/mL), MwB3, MwB2, and MwB1 had recorded rates of inhibition of about 38%, 45%, and 10%, respectively. To better understand in which way and at which stage of the virus life cycle the extracts acted, further tests were conducted. When the extracts were added directly to the viral suspension for 1 h (virus pre-treatment test, Figure 2B,D) and the mixture was diluted on the cell monolayer, an increase in the inhibitory effect was observed. Indeed, MwS3 and MwS2 (Figure 2B) under this experimental condition exhibited an IC_50_ at 10 µg/mL and 22 µg/mL, respectively. For the extracts MwB3 and MwB2 (Figure 2D), the percentage of inhibition was slightly increased in comparison to those observed in the co-treatment assay, with 42% and 50% inhibition of infection at 200 µg/mL, respectively.

**Figure 3:**
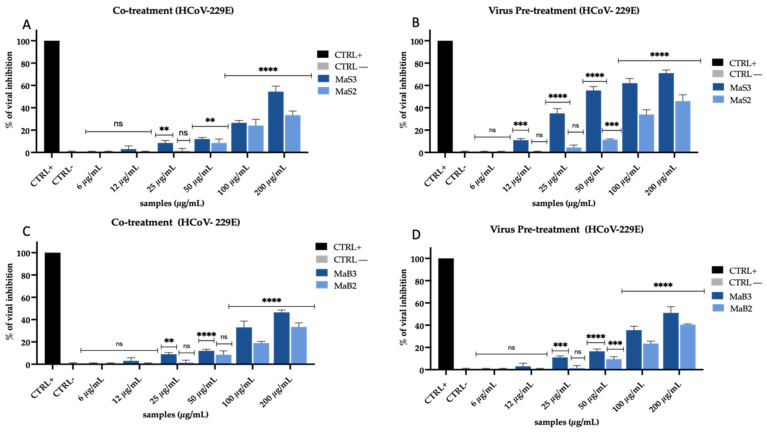
Antiviral activity of *M. oleifera* extracts against HCoV-229E. Co-treatment test (**A**) and virus pre-treatment (**B**) of MaS extracts; co-treatment (**C**) and pre-treatment test (**D**) of MaB extracts. Algal extract at 50 µg/mL was used as a positive control (CTRL+), while infected and untreated cells represented negative control (CTRL−). Two-way ANOVA was used for statistical analysis. The Dunnett test was used for multiple comparisons. **** *p* < 0.0001, *** *p* ≤ 0.0002, ** *p* < 0.0021, * *p* < 0.0332, ns: not statistically significant.

Then, we evaluated the action of the same extracts obtained by maceration, observing that it was quite different (Figure 3).

MaS extracts were found to be less effective than MwS extracts. In detail, MaS3 and MaS2 recorded 55% and 33% inhibition at the highest concentration (200 µg/mL) in co-treatment test (Figure 3A). In virus pre-treatment (Figure 3B), their antiviral activity increased but remained lower than the extracts in Figure 2, with an inhibition of 70% (MaS3) and 55% (MaS2) at 200 µg/mL. MaB extracts showed a similar trend as before, with a mild activity in co-treatment (Figure 3C) for MaB3 and MaB2 (46% and 33%, respectively, at 200 µg/mL), while it resulted improved in virus pre-treatment (Figure 3D), with 50% and 40% inhibition, respectively, at 200 µg/mL.

For both types of extraction and both cultivars, no inhibition occurred when cells were first pretreated with extracts and then infected with the virus (cell pre-treatment), nor when the virus was first added to the cells and, subsequently, cells were treated with the extracts (post-treatment). These results suggested an immediate action of the extracts aimed at the early stages of infection, namely attachment and penetration into the host cells. In addition, the obtained results point to quali-quantitative differences in the extract composition as the cause of the observed differences in the antiviral performance against HCoV-229E. The IC50 calculated for each extract against HCoV-229E by performing a virus pre-treatment scheme, associated with their CC50 and SI, are reported in Supplementary Materials (Table S1).

#### 3.2.2. Antiviral Activity against MeV

**Figure 4:**
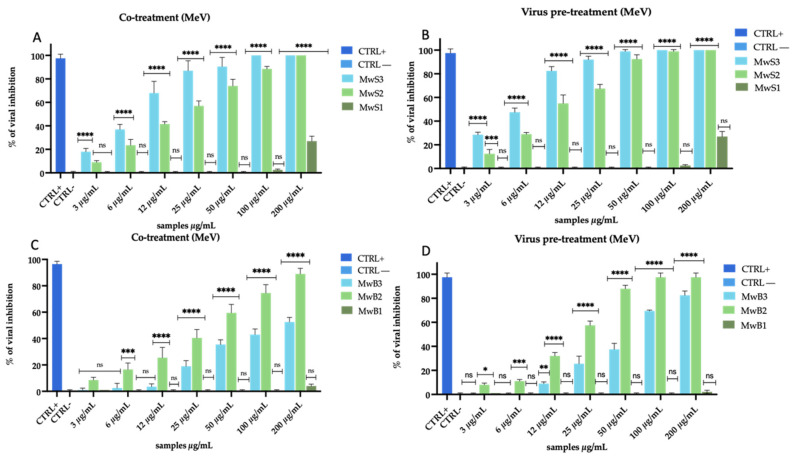
Antiviral activity of *M. oleifera* extracts against MeV. Co-treatment test (**A**) and virus pre-treatment (**B**) of MwS extracts; co-treatment test (**C**) and virus pre-treatment test of MwB extracts (**D**). The peptide AR-23 at 25 µg/mL was used as a positive control (CTRL+), while infected and untreated cells represented negative control (CTRL−). Two-way ANOVA was used for statistical analysis. The Dunnett test was used for multiple comparisons. **** *p* < 0.0001, *** *p* ≤ 0.0002, ** *p* < 0.0021, * *p* < 0.0332, ns: not statistically significant.

**Figure 5:**
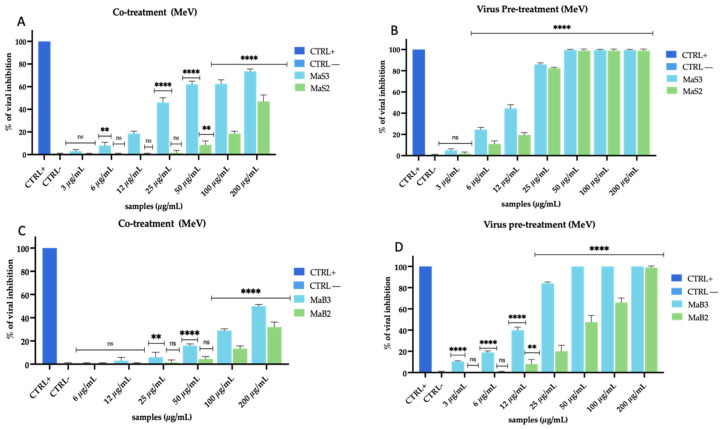
Antiviral activity of *M. oleifera* extracts against MeV. Co-treatment test (**A**) and virus pre-treatment (**B**) of MaS extracts; co-treatment (**C**) and pre-treatment test (**D**) of MaB extracts. The peptide AR-23 at 25 µg/mL was used as a positive control (CTRL+), while infected and untreated cells represented negative control (CTRL−). Two-way ANOVA was used for statistical analysis. The Dunnett test was used for multiple comparisons. **** *p* < 0.0001, ** *p* < 0.0021, ns: not statistically significant.

We selected an additional virus to study the mode of action of the extracts. Therefore, the same experiments described above were conducted against the MeV, using Vero/hSLAM cells. Under all the tested conditions, the antiviral activity was similar to that detected for HCoV-229E, but a greater inhibitory effect was recorded for all extracts. They displayed antiviral activity against MeV when the cells were co-treated with extracts and virus (Figure 4A,C and Figure 5A,C), and when the virus was pre-incubated with extracts (Figure 4B,D and Figure 5B,D), as it was observed previously.

In detail, MwS extracts recorded a strong inhibition of infection in co-treatment (Figure 4A): MwS3 exhibited an IC_50_ at 9 µg/mL, MwS2 at 21 µg/mL, while, on the contrary, and similarly to what was observed with HCoV-229E, MwS1 did not show any antiviral effect. Pre-treating the virus with extracts caused an improvement in their activity (Figure 4B): MwS3 reached an IC_50_ at 6 µg/mL, while MwS2 at 10 µg/mL. Likewise, MwB extracts also exhibited activity in the same treatments (Figure 4C,D). Surprisingly, the inhibition rates were higher than those obtained against HCoV-229E but still remained lower than those obtained with the Salento cultivar (Figure 2). In co-treatment (Figure 4C), MwB3 and MwB2 showed inhibitions of 35% and 60% at 50 µg/mL and reached 38% and 88% in virus pre-treatment (Figure 4D).

The results obtained against MeV with extracts obtained by maceration were noteworthy (Figure 5).

Unlike what happened with HCoV-229E, we observed a far greater effect in virus pre-treatment for both cultivars while, on the other side, the action was rather weak in the co-treatment. In detail, MaS3 and MaS2 reached 85% and 80% until 25 µg/mL, respectively (Figure 5B); MaB3 also showed 85% inhibition at 25 µg/mL, while MaB2 reached 50% inactivation at 50 µg/mL (Figure 5D). However, also in this case, no inhibition occurred in cell pre-treatment and post-treatment, indicating that there was no interaction with the cell membrane and/or receptors present on it and no interference with viral replication. Overall, the two types of extraction and the extracts of both cultivars showed high rates of inhibition in co-treatment and virus pre-treatment against MeV. The interesting thing that we noticed was a greater action of MwS extracts. For this reason, we investigated the action of these extracts on the human lung cells A549 to study whether the antiviral activity was comparable to that observed on Vero cells. Surprisingly, the activity was confirmed and even implemented when the infection was performed on the lung cells representing the main infection site of the studied viruses (Figure S1, Supplementary Materials).

#### 3.2.3. Temperature-Shift Assays to Assess Mode of Action

**Figure 6:**
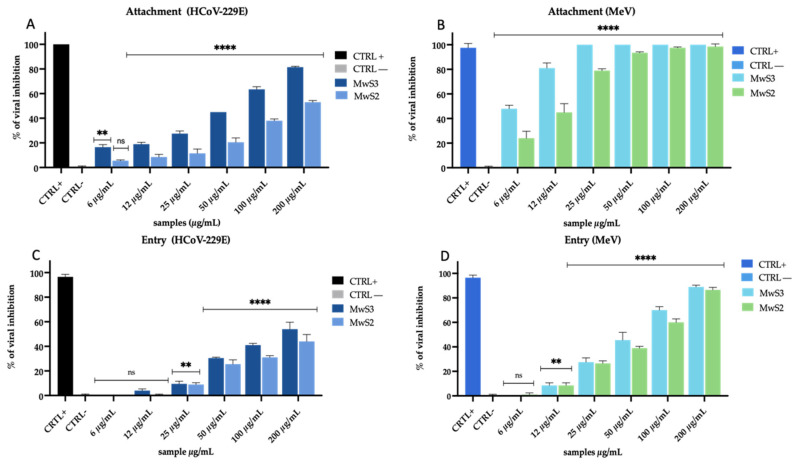
Temperature shift assays. Attachment and entry tests against HCoV-229E (**A**–**C**) and MeV (**B**–**D**). Positive controls were algal extract at 50 µg/mL (HCoV-229E), and peptide AR-23 at 25 µg/mL (MeV). Infected and untreated cells represented negative control (CTRL−). Two-way ANOVA was used for statistical analysis. The Dunnett test was used for multiple comparisons. **** *p* < 0.0001, ** *p* < 0.0021, ns: not statistically significant.

Therefore, in further investigating the mode of action, we used MwS extracts to study the early stages of the viral cycle, i.e., attachment and entry in the host cell [32] (Figure 6). In the attachment assay, the cells were pre-cooled at 4 °C and then co-treated with extracts and virus for 1 h. The low temperature favors the binding between viral and cellular membranes but does not allow the entry of the virus inside the cell. The results indicated an inhibition of MwS3 and MwS2 against HCoV-229E of 60% and 38% up to 100 µg/mL (Figure 6A). The activity was higher against MeV with a total inhibition at the highest concentrations, and 80% and 50% inhibition up to 12 µg/mL (Figure 6B). In the entry assay, the cells were first infected at 4 °C and then treated with extracts at 37 °C for 1 h. At low temperatures, the virus binds to the membrane, so the subsequent shift to 37 °C immediately enables the virus to enter the cell. Here again, MwS3 and MwS2 presented 40% and 35% inhibition up to 100 µg/mL (Figure 6C), while the same results were detected at the lower concentration of 50 µg/mL against MeV (Figure 6D).

### 3.3. Qualitative and Quantitative Analysis of the Main Bioactive Compounds in the M. oleifera Extracts

**Figure 7:**
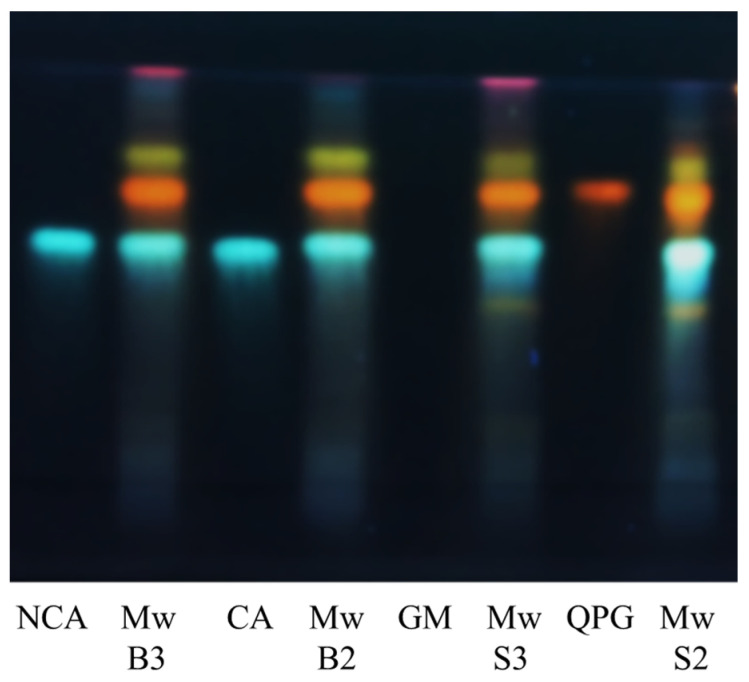
HPTLC profile of *M. oleifera* extracts obtained under microwave irradiation and four reference standards sprayed with NP/PEG reagent; MwB2 and MwB3: *M. oleifera* Barletta extracted with 50% EtOH and 70% EtOH, respectively; MwS2 andMwS3: *M. oleifera* Salento extracted with 50% EtOH and 70% EtOH, respectively; CA: chlorogenic acid; NCA: neochlorogenic acid; QPG: quercetin 3-*O*-β-d-glucopyranoside; GM: glucomoringin; solvent: ethyl acetate/formic acid/acetic acid/water (100:11:11:26); λ: 366 nm.

The bioactive extracts were analyzed through HPTLC and HPLC-DAD to possibly obtain more insights into compounds putatively responsible for the observed biological results. A preliminary qualitative analysis was performed through the HPTLC technique, and the obtained results are reported in Figure 7; chlorogenic and neochlorogenic acids (CA and NCA, respectively), quercetin 3-*O*-β-d-glucopyranoside (QGP), and glucomoringin (GM) were used as standard reference compounds, with GM being an uncommon member of glucosinolate group peculiar for the *Moringaceae* family. The presence of these secondary metabolites was confirmed in the analyzed extracts, except for GM, which was not detectable through the HPTLC analysis.

The extracts were then analyzed by HPLC to determine the bioactive compound content and the relative chromatograms are described in Figures S2–S9 (Supplementary Materials). With the aim of comparing the two analyzed *M. oleifera* varieties, no significant difference in both the chlorogenic and neochlorogenic acids amounts in MwS2 and MwB2 was observed. On the contrary, greater amounts of both acids were extracted from MwS3 in comparison with MwB3, particularly in the case of neochlorogenic acid. With regard to quercetin 3-*O*-β-d-glucopyranoside, a greater abundance of this compound was observed in MwB, regardless of the adopted extraction solvent. Considering that the MwB extracts were always less active than MwS ones, the antiviral activities do not seem directly related to the quercetin 3-*O*-β-d-glucopyranoside content. On the contrary, the glucomoringin content was higher in MwS3 than in Mws2. Focusing attention on the solvents’ extracting power, EtOH 50% gave a greater amount of both acids from MwB and of chlorogenic acid only from MwS, while EtOH 70% gave a higher neochlorogenic acid level in the MwS extract, as stated above. Finally, the water percentage in the hydroalcoholic mixture did not affect the glucomoringin extraction from MwB, unlike what happened for MwS. The latter showed a 0.60 µg/mL content of the glucosinolate in MwS3, the highest amount reached in this study. Summing up these findings, the higher content of glucomoringin and neochlorogenic acid in MwS3 probably contributed to its greater antiviral activities.

## 4. Discussion

Respiratory viruses are one of the main causes of illness in the human population, causing the development of infections in the respiratory tract and, in some cases, difficulties that can lead to acute respiratory distress syndrome (ARDS) [33,34]. In the present study, we demonstrated the antiviral activity of *M. oleifera* leaves obtained from two different Apulian varieties and underwent an eco-friendly and sustainable extraction procedure previously developed in our laboratories [31]. Both aqueous and hydroalcoholic extracts were prepared through the modern and green MAE procedure that allows the reduction of time, energy, and solvent consumption compared to conventional extraction methods, such as maceration [35]. Furthermore, the MAE technique guarantees the minimal thermal degradation of biomolecules even when working at high temperatures, since a shorter extraction time reduces the risk of decomposition and oxidation of phytochemicals, as demonstrated in our previous studies [36,37]. Despite a slightly lower extraction yield obtained with MAE (17–19%) in comparison with maceration (21–23%), this study has confirmed that microwave irradiation improves the extraction process ensuring both a remarkable reduction in extraction times and extracts endowed with lower cytotoxicity and higher antiviral activity against HCoV-229E and MeV, two viruses involved in respiratory infections [38,39].

Several beneficial properties have been reported by *M. oleifera*, including its usage as food for its high nutritional content [40,41]. Some studies have focused on the antiviral properties of the extracts of *M. oleifera*, but the components mainly responsible for the activity remain unclear [40].

Our results obtained from HPLC analyses revealed mainly the presence of some secondary metabolites in the active extracts, such as chlorogenic, neochlorogenic acid, and glucomoringin, which could be responsible for the antiviral activity. Particularly remarkable were the results obtained with MwS3 and MwS2 in virus pre-treatment condition. We demonstrated that MwS3 and MwS2 reached a 50% inhibition at 10 µg/mL and 22 µg/mL against HCoV-229E, respectively; additionally, they induced a 50% viral reduction at 6 µg/mL and 10 µg/mL, respectively, when used against MeV. Moreover, higher levels of neochlorogenic acid and glucomoringin were observed in the extract MwS3 compared to MwS2, and this could explain the enhanced activity. The anti-SARS-CoV-2 activity of some of the identified metabolites has recently been reported [20,42,43,44]. Notably, based on our results, the two *M. oleifera* varieties under evaluation showed different antiviral activities, with the Salento variety being generally more active than the Barletta one against both the studied viruses (HCoV-229E and MeV). Furthermore, the best results were obtained when 70% EtOH was used as the extraction solvent and MAE as the extraction procedure, thus highlighting the great advantages of this innovative extraction technique with respect to maceration. A similar variability in activity profiles, depending on experimental settings and different varieties, was previously reported in similar studies on the same or different *M. oleifera* varieties [31].

Lipipun et al. showed that *M. oleifera* leaf extracts had antiviral activity against HSV-1 with IC_50_ at 100 µg/mL, a much higher concentration than those observed in our study [45]. Other studies on the activity of leaves of this plant have shown its efficacy against hepatitis B virus (HBV) and human immunodeficiency virus (HIV) [26,46]. However, as far as we know, there have been no reported activities against HCoV-229E and MeV to date. Only computational studies and molecular docking against human coronaviruses have been conducted evidencing the interaction of *M. oleifera* bioactive components with the Mpro protein, responsible for SARS-CoV-2 replication.

We found that our extracts were active in these early stages of infection, preventing the attachment and the entry of both viruses into the host cell. In detail, these steps occur thanks to the interactions between viral glycoproteins and cellular receptors that lead to the fusion of the two membranes [47]. HCoV-229E has four structural proteins, i.e., S, M, N, and E, and the glycoprotein S binds to the CD13 receptor to initiate infection [48]. On the other side, the input of MeV to initiate the infection is mediated by two surface glycoproteins, namely H, which is the antireceptor binding to CD150 on the cell surface, and F, which promotes the membranes’ fusion [49]. We observed a strong inhibition of viral attachment and entry, especially against MeV, and almost double at 100 µg/mL compared to HCoV-229E, and we hypothesize that the different activity could be due to a different affinity for the viral glycoproteins. In addition, negative results from the cell pre-treatment and post-treatment assays indicated that extracts had no effect on the cell surface and inside the infected cell, thus preventing some intracellular mechanisms, such as viral replication. On the contrary, it is known that *M. oleifera* seed extracts had an inhibitory effect against influenza A virus in both cell pre-treatment and post-treatment experiments, but this divergence could be explained by the different viruses studied and by the extract derived from different sources; in fact, seeds are richer in oils than leaves [50]. Finally, Zhang et al. reported the antiviral activity of the aqueous leaf extract of *M. oleifera* against a member of the *Coronaviridae* family, the swine epidemic diarrhea virus (PEDV). The inhibitory effect was observed in the late stage of infection at the very high concentration of 5000 µg/mL [51].

## 5. Conclusions

Natural plant products are an important resource for their diverse biological activities. In the present study, we demonstrated that the leaves of *M. oleifera*, derived from two Apulian cultivars (Salento and Barletta) and extracted with the MAE technique, were capable of preventing viral infection of HCoV-229E and MeV, without showing significant cytotoxicity on in vitro cellular models. In particular, the extract obtained with 70% of EtOH (Salento cultivar), exhibited a higher percentage of inhibition in the early stages of viral infection. This extract had higher levels of chlorogenic acid, neochlorogenic, and glucomoringin, which are probably responsible for this greater efficacy. However, we cannot exclude that the antiviral effect is related to other unidentified constituents. Therefore, the next steps of our work will focus on the fractionation of the raw extracts and characterization of the main components by liquid chromatography coupled to high-resolution mass spectrometry (LC-HRESIMS) and liquid chromatography coupled to tandem spectrometry (LC-MS/MS). In addition, we aim to evaluate the antiviral activity of the individual compounds on human cell lines of the lung tract to mimic the real infection environment.

## Figures and Tables

**Table 1:** General information on *M. oleifera* leaf extracts.

Cultivar	H_2_O		50% EtOH		70% EtOH	
	MAE Extraction	Maceration Extraction	MAE Extraction	Maceration Extraction	MAE Extraction	Maceration Extraction
Salento	MwS1	-	MwS2	MaS2	MwS3	MaS3
Barletta	MwB1	-	MwB2	MaB2	MwB3	MaB3

**Table 2:** Percentage (%) of yields obtained for each extract.

Extract	Yield (%)
MwS1	27.4%
MwB1	25.0%
MwS2	17.2%
MwB2	17.9%
MaS2	21.0%
MaB2	21.6%
MwS3	18.4%
MwB3	19.0%
MaS3	22.4%
MaB3	23.2%

## Data Availability

The data presented in this study are available on request from the corresponding author. The authors can confirm that all relevant data are included in the article.

## Supplementary Materials

The following supporting information can be downloaded at: https://www.mdpi.com/article/10.3390/v16081199/s1, Figure S1: Antiviral activity of *M. oleifera* extracts (MwS) on A549 cell line; Figure S2: HPLC-PDA chromatograms recorded at 327 nm of MwS2, MwB2 and standards; Figure S3: HPLC-PDA chromatograms recorded at 327 nm of MwS3, MwB3 and standards; Figure S4: HPLC-PDA chromatograms recorded at 327 nm of MwS2, MwB2 and standards; Figure S5: HPLC-PDA chromatograms recorded at 327 nm of MwS3, MwB3 and standards; Figure S6: HPLC-PDA chromatograms recorded at 230 nm of MwS2, MwB2 and standards; Figure S7: HPLC-PDA chromatograms recorded at 230 nm of MwS3, MwB3 and standards; Figure S8: HPLC-PDA chromatograms recorded at 254 nm of MwS2, MwB2 and standards; Figure S9: HPLC-PDA chromatograms recorded at 254 nm of MwS3, MwB3 and standards; Table S1: CC50, IC50 and SI for each extract against HCoV-229E; Table S2: CC50, IC50 and SI for each extract against MeV.
