# Supplementary material for: Antiviral Properties of Moringa oleifera Leaf Extracts against Respiratory Viruses
Source: Viruses. 2024 Jul 25;16(8):1199. doi: 10.3390/v16081199 (PMC11359668; doi:10.3390/v16081199)
Supplement: Supplementary file 1 [file viruses-16-01199-s001.zip › viruses-3075803-supplementary.pdf]

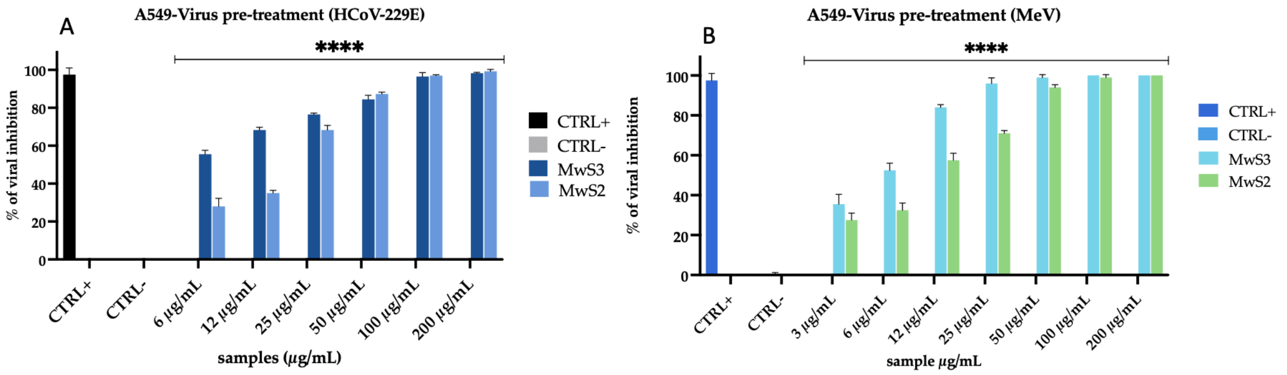

Figure S1. Antiviral activity of *M. oleifera* extracts (MwS) on A549 cell line. Virus pre-treatment against HCoV-229E **(A)** and MeV **(B)** was performed. Algal extract at 50 µg/mL (for HCoV-229E) and the peptide AR-23 at 25 µg/mL (for MeV) were used as positive control (CTRL+), while infected and untreated cells represented negative control (CTRL –). Two-way ANOVA was used for statistical analysis. The Dunnett test was used for multiple comparisons. **** p < 0.0001.

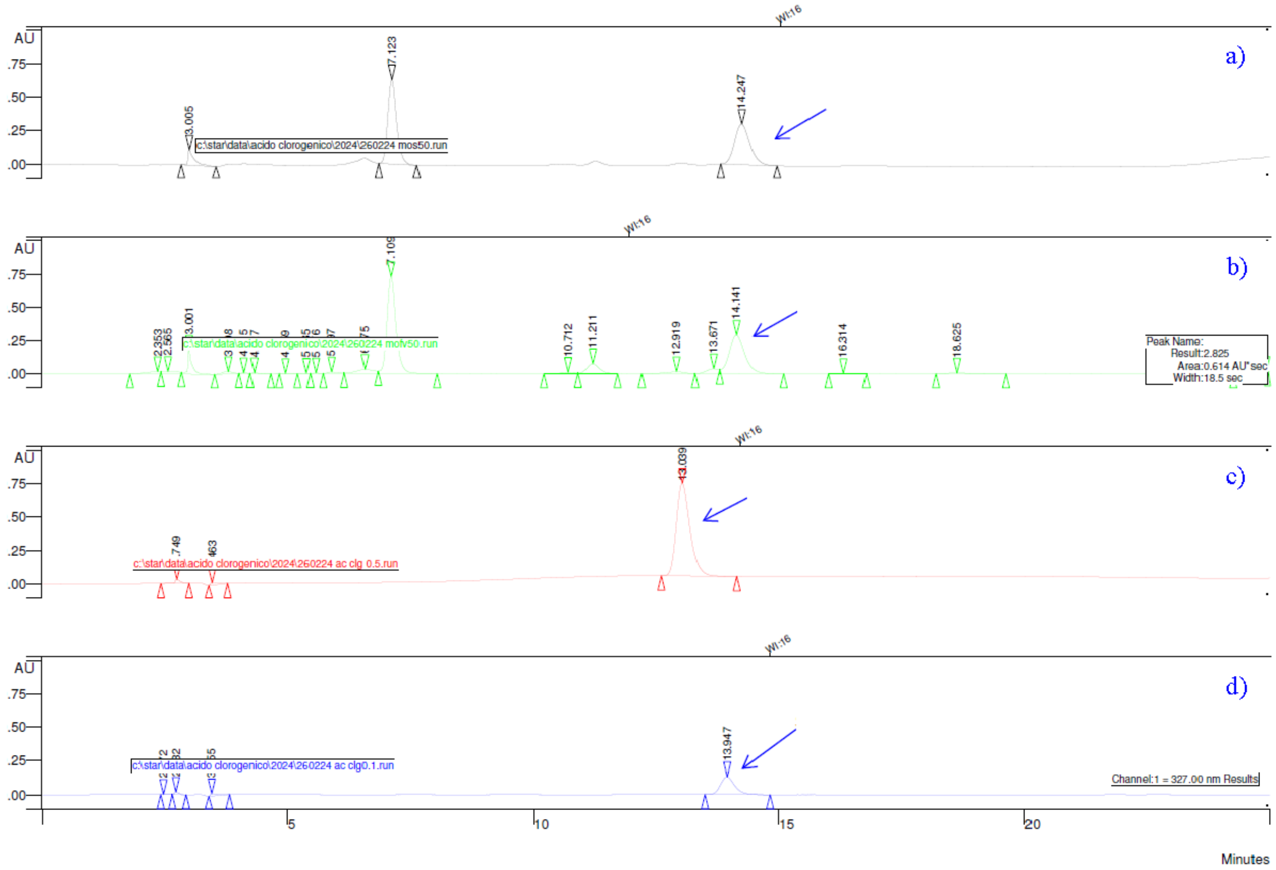

Figure S2. HPLC-PDA chromatograms recorded at 327 nm of MwS2, MwB2 and standards. a) MwS2 extract, b) MwB2 extract, c) standard: chlorogenic acid 0.5 mg/mL; d) standard: chlorogenic acid 0.1 mg/mL. Column Luna Phenomenex C18 (250 × 4.6) 5 µm, mobile phase ACN/H_3PO_4 0.5 % (11.5/88.5), injection volume 10 µL, flow rate 1 mL/min, column temperature 25 °C.

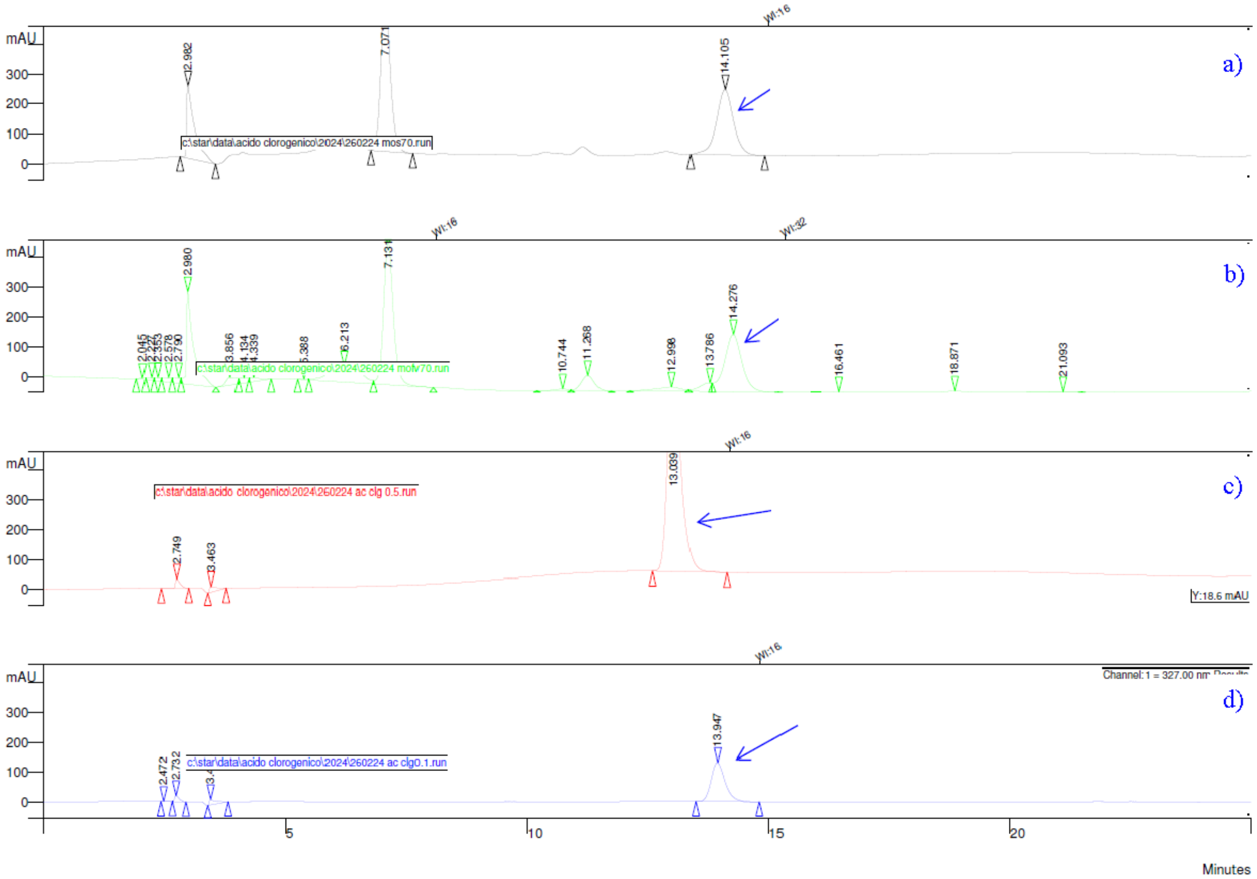

Figure S3. HPLC-PDA chromatograms recorded at 327 nm of MwS3, MwB3 and standards. a) MwS3 extract, b) MwB3 extract, c) standard: chlorogenic acid 0.5 mg/mL; d) standard: chlorogenic acid 0.1 mg/mL. Column Luna Phenomenex C18 (250 × 4.6) 5 µm, mobile phase ACN/H_3PO_4 0.5 % (11.5/88.5), injection volume 10 µL, flow rate 1 mL/min, column temperature 25 °C.

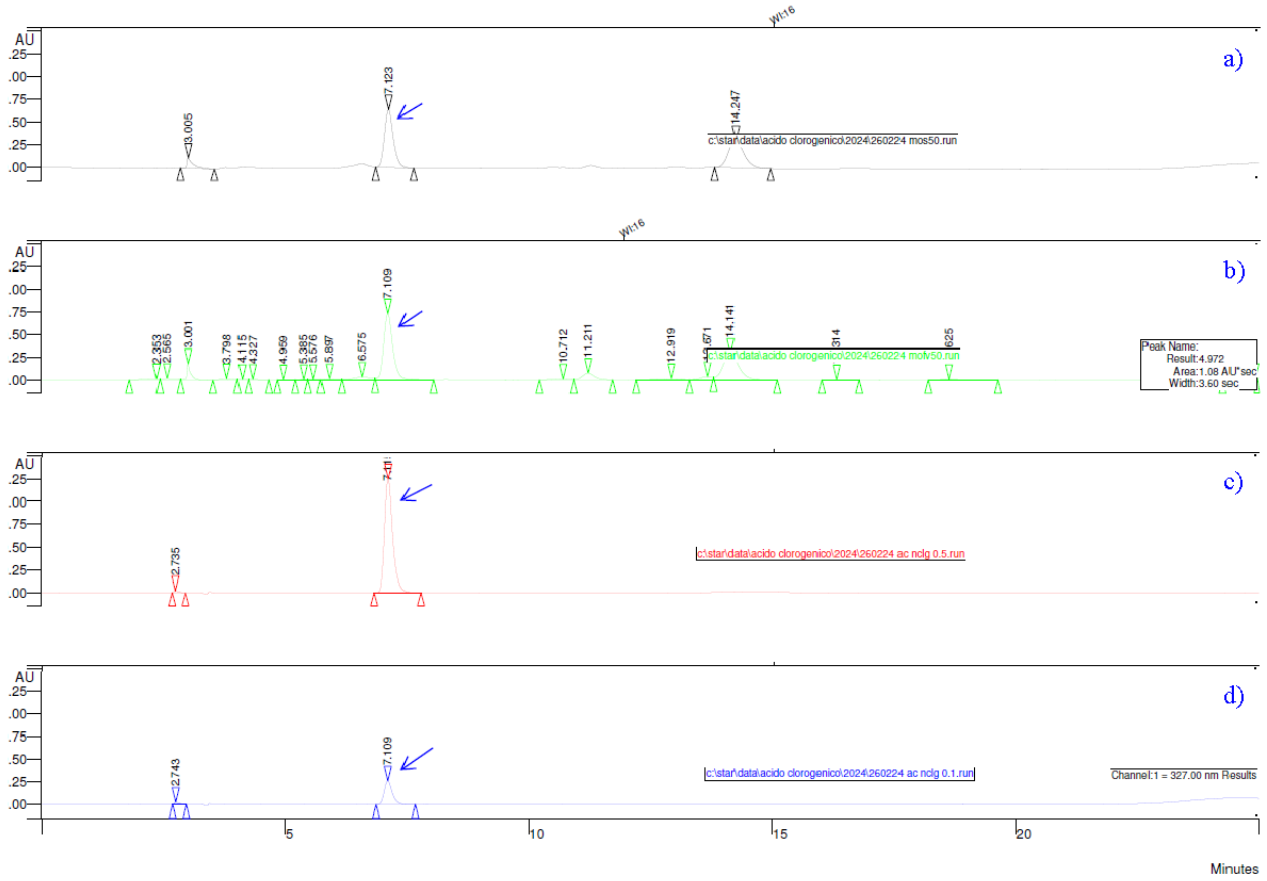

Figure S4. HPLC-PDA chromatograms recorded at 327 nm of MwS2, MwB2 and standards. a) MwS2 extract, b) MwB2 extract, c) standard: neochlorogenic acid 0.5 mg/mL; d) standard: neochlorogenic acid 0.1 mg/mL. Column Luna Phenomenex C18 (250 × 4.6) 5 µm, mobile phase ACN/H_3PO_4 0.5 % (11.5/88.5), injection volume 10 µL, flow rate 1 mL/min, column temperature 25 °C.

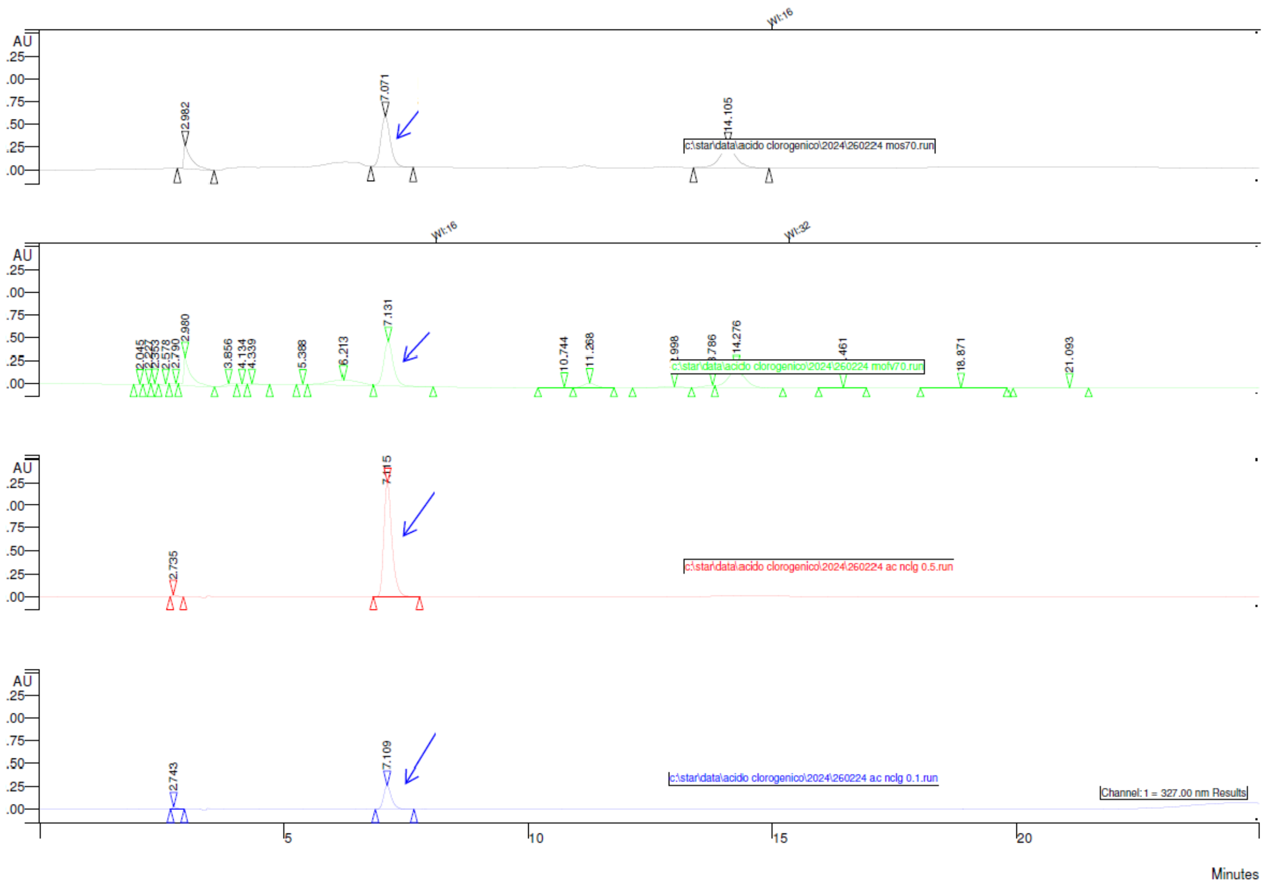

Figure S5. HPLC-PDA chromatograms recorded at 327 nm of MwS3, MwB3 and standards. a) MwS3 extract, b) MwB3 extract, c) standard: neochlorogenic acid 0.5 mg/mL; d) standard: neochlorogenic acid 0.1 mg/mL. Column Luna Phenomenex C18 (250 × 4.6) 5 µm, mobile phase ACN/H_3PO_4 0.5 % (11.5/88.5), injection volume 10 µL, flow rate 1 mL/min, column temperature 25 °C.

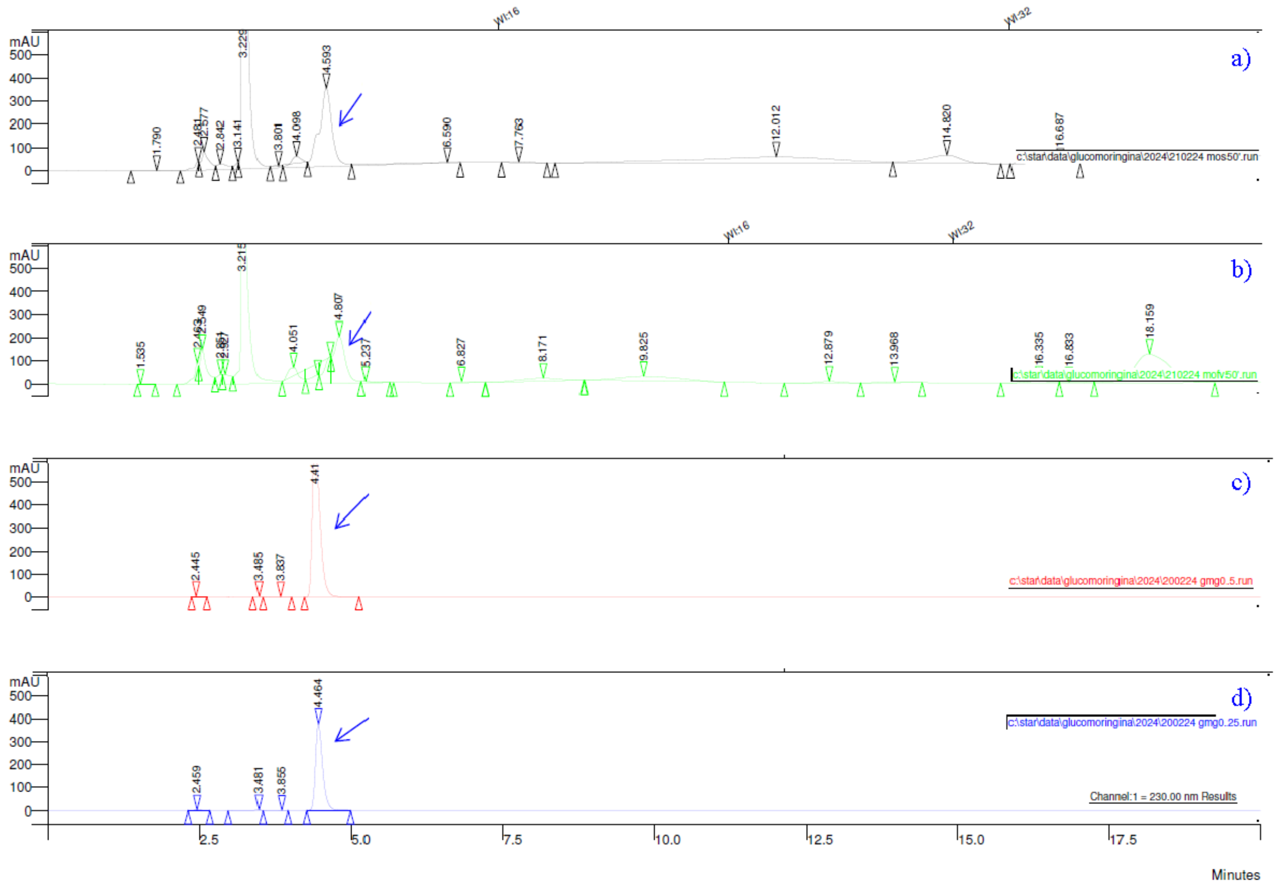

Figure S6. HPLC-PDA chromatograms recorded at 230 nm of MwS2, MwB2 and standards. a) MwS2 extract, b) MwB2 extract, c) standard: glucomoringin 0.5 mg/mL; d) standard: glucomoringin 0.25 mg/mL. Column Luna Phenomenex C18 (250 × 4.6) 5 µm, mobile phase TFA 0.1%/ACN (95/5), injection volume 10 µL, flow rate 1 mL/min, column temperature 30 °C.

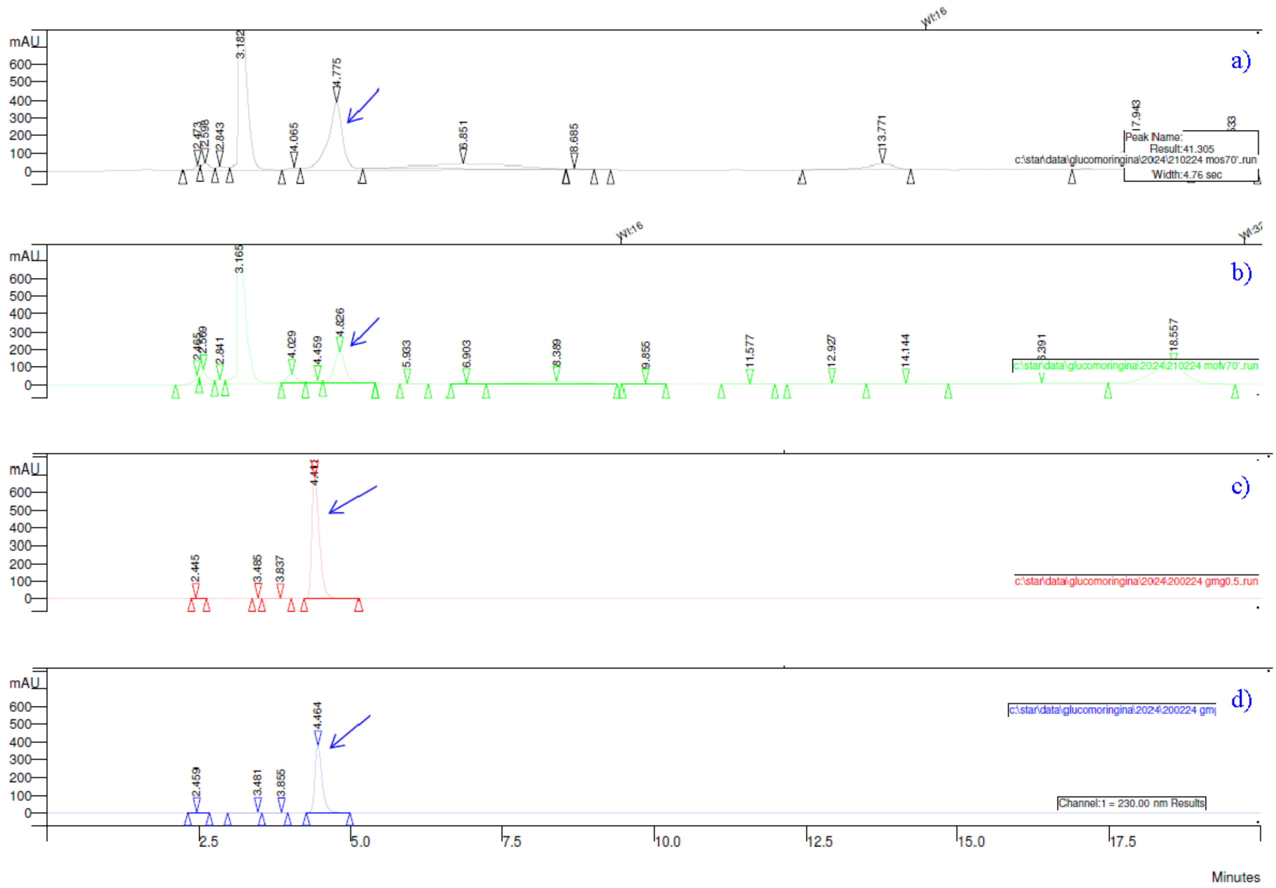

Figure S7. HPLC-PDA chromatograms recorded at 230 nm of MwS3, MwB3 and standards. a) MwS3 extract, b) MwB3 extract, c) standard: glucomoringin 0.5 mg/mL; d) standard: glucomoringin 0.25 mg/mL. Column Luna Phenomenex C18 (250 × 4.6) 5 µm, mobile phase TFA 0.1%/ACN (95/5), injection volume 10 µL, flow rate 1 mL/min, column temperature 30 °C.

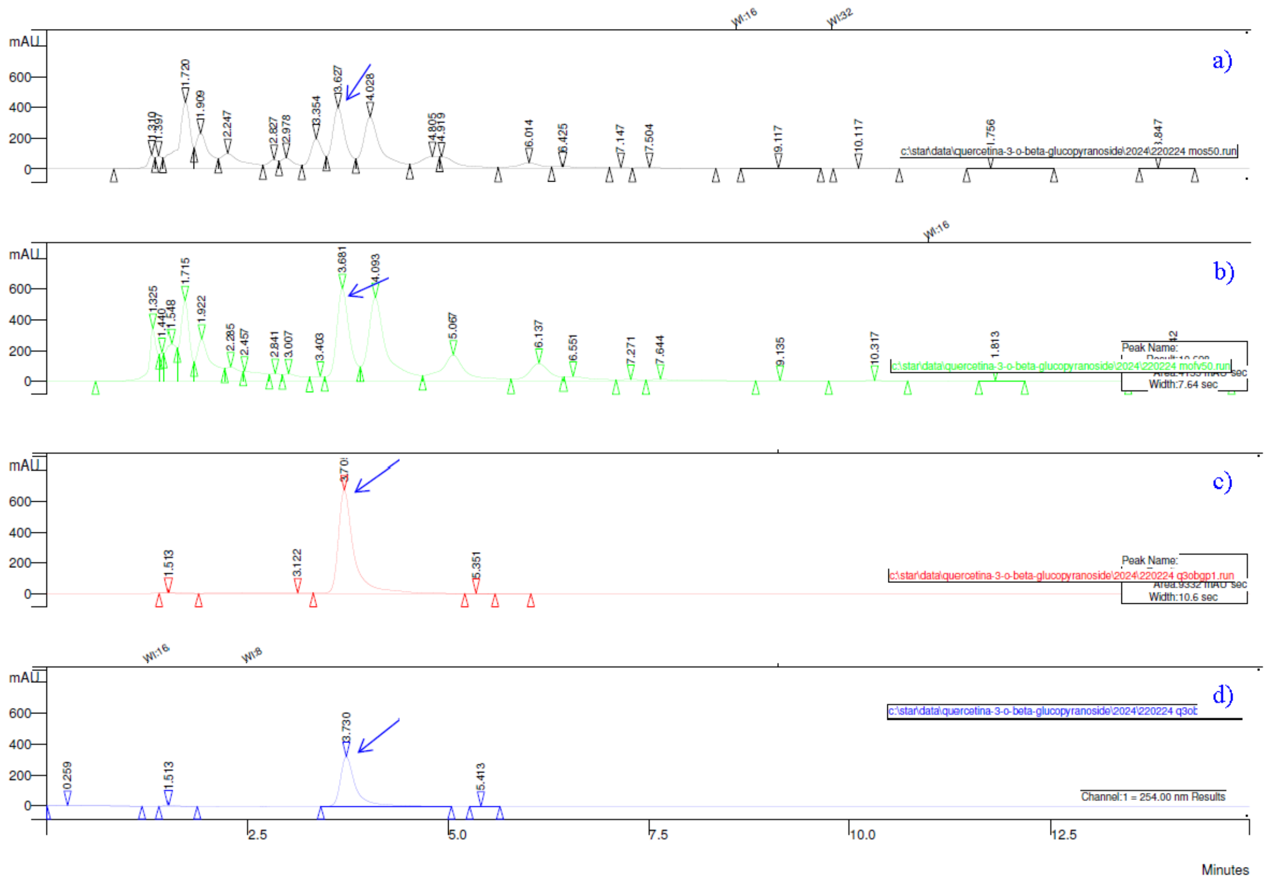

Figure S8. HPLC-PDA chromatograms recorded at 254 nm of MwS2, MwB2 and standards. a) MwS2 extract, b) MwB2 extract, c) standard: quercetin 3-*O*-β-D-glucopyranoside 1 mg/mL; d) standard: quercetin 3-*O*-β-D-glucopyranoside 0.5 mg/mL. Column Luna Phenomenex C18 (150 × 4.6) 5 μm, mobile phase H_3PO_4 0.3%/MeOH/ACN (55/35/10), injection volume 5 μL, flow rate 1 mL/min, wavelength 254 nm, column temperature 30 °C.

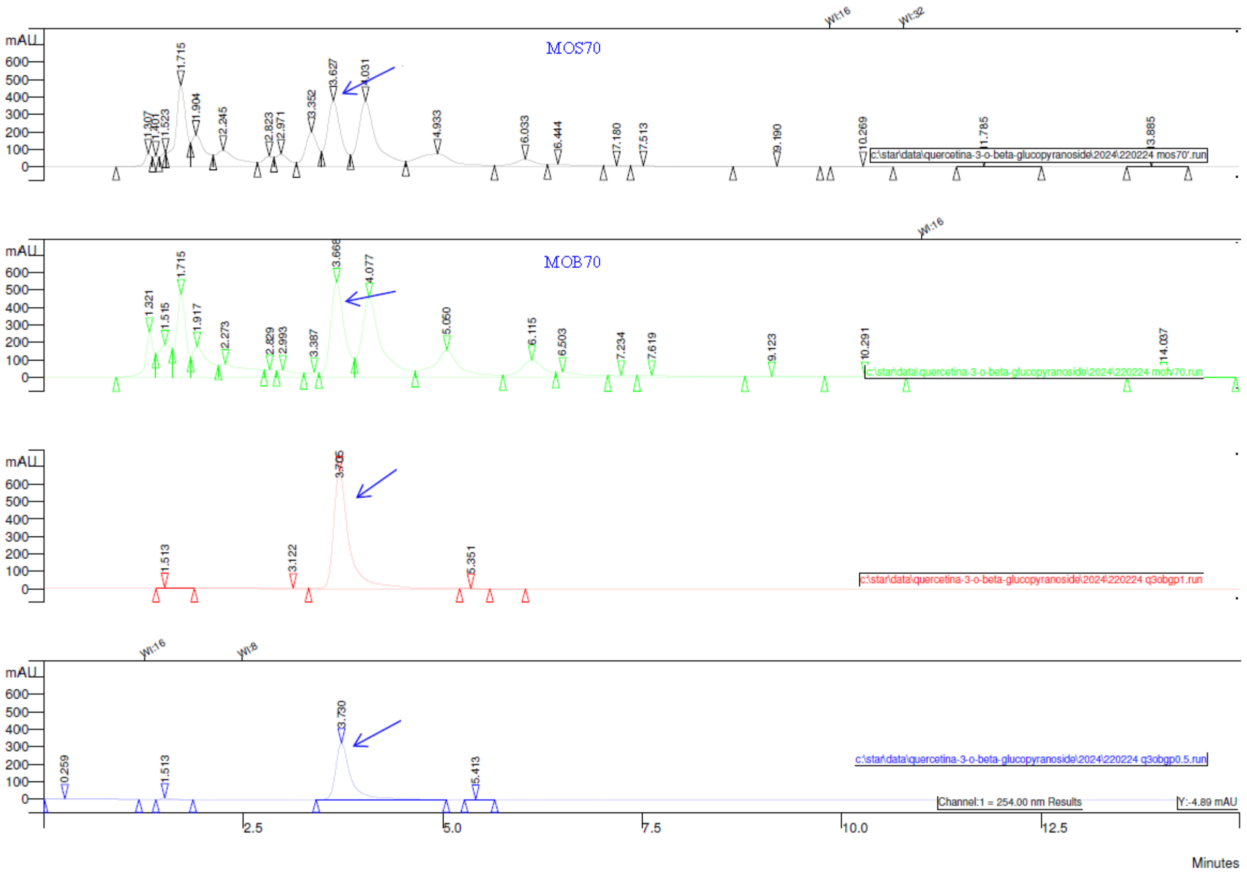

Figure S9. HPLC-PDA chromatograms recorded at 254 nm of a) MwS3 extract, b) MwB3 extract, c) standard: quercetin 3-*O*-β-D-glucopyranoside 1 mg/mL; d) standard: quercetin 3-*O*-β-D-glucopyranoside 0.5 mg/mL. Column Luna Phenomenex C18 (150 × 4.6) 5 μm, mobile phase H_3PO_4 0.3%/MeOH/ACN (55/35/10), injection volume 5 μL, flow rate 1 mL/min, column temperature 30 °C.

Table S1. CC50, IC50 and SI for each extract against HCoV-229E. CC50 has been calculated on Vero CCL-81 cells. IC50 has been obtained by virus pre-treatment data.

Extract	CC50 (μg/mL)	IC50 (μg/mL)	SI
MwS1	> 400	-	-
MwB1	> 400	-	-
MwS2	> 400	22	18.2
MwB2	> 400	200	2
MaS2	300	250	1.2
MaB2	> 400	250	1.6

MwS3	> 400	10	40
MwB3	> 400	250	1.6
MaS3	> 400	45	8.9
MaB3	> 400	200	2

Table S2. CC50, IC50 and SI for each extract against MeV. CC50 has been calculated on Vero/hSLAM cells. IC50 has been obtained by virus pre-treatment data.

Extract	CC50 (µg/mL)	IC50 (µg/mL)	SI
MwS1	> 400	-	-
MwB1	> 400	-	-
MwS2	> 400	10	40
MwB2	> 400	20	20
MaS2	80	20	4
MaB2	> 400	50	8
MwS3	> 400	6	66.7
MwB3	> 400	70	5.7
MaS3	300	10	3
MaB3	> 400	10	40
